# Supplementary figures and images for: The ESCRT machinery regulates retromer-dependent transcytosis of septate junction components in Drosophila
Source: eLife. 2020 Dec 30;9:e61866. doi: 10.7554/eLife.61866 (PMC7848756; doi:10.7554/eLife.61866)

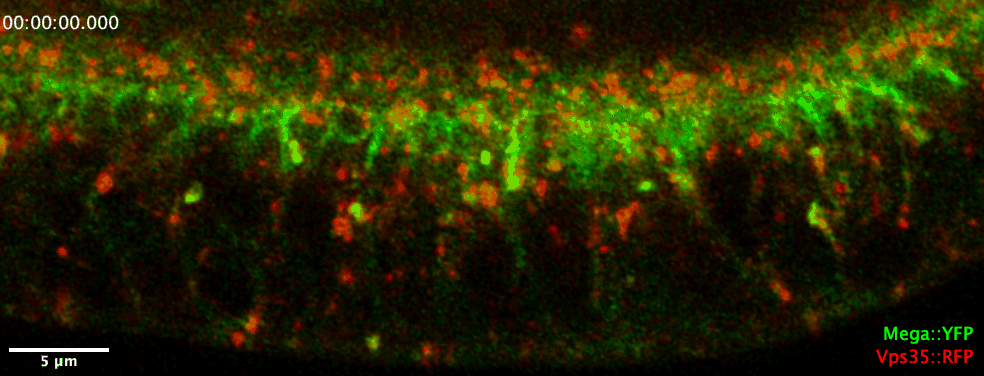

Supplement: Supplementary file 6 [file elife-61866-video1.gif]
